# Metyrosine-associated endocrinological changes in pheochromocytoma and paraganglioma

**DOI:** 10.1530/EO-23-0006

**Published:** 2023-08-30

**Authors:** Yuko Matsuo, Kenji Ashida, Ayako Nagayama, Kanoko Moritaka, Mizuki Gobaru, Junichi Yasuda, Naoyuki Ogasawara, Hirofumi Kurose, Katsuaki Chikui, Shimpei Iwata, Yukihiro Inoguchi, Nao Hasuzawa, Seiichi Motomura, Tsukasa Igawa, Masatoshi Nomura

**Affiliations:** 1 Division of Endocrinology and Metabolism, Department of Internal Medicine, Kurume University School of Medicine, Asahi-machi, Kurume, Fukuoka, Japan; 2 Department of Urology, Kurume University School of Medicine, Asahi-machi, Kurume, Fukuoka, Japan

**Keywords:** pheochromocytoma, paraganglioma, dopamine, diabetes mellitus, prolactin

## Abstract

**Objective:**

Metyrosine (alpha-methyl-para-tyrosine) effectively reduces catecholamine levels in patients with pheochromocytoma/paraganglioma. However, improvements in physiological and metabolic parameters and changes in endocrine function associated with metyrosine administration should be validated in comparison to surgery. This study was performed to confirm the effects of metyrosine on the physiological, metabolic, and endocrinological functions of patients with pheochromocytoma/paraganglioma in the perioperative period.

**Design:**

This retrospective cohort study was performed at a single university hospital.

**Methods:**

We included ten patients with pheochromocytoma/paraganglioma who received oral metyrosine after α-blocker therapy and consecutive surgeries. Urinary catecholamine metabolite levels and other clinical parameters were evaluated before and after metyrosine administration, and 1 week after surgery.

**Results:**

The mean age was 53.1 ± 16.1 years. Of the ten participants (four men and six women), nine had pheochromocytoma and one had paraganglioma. The median maximum metyrosine dose was 750 mg/day. Urinary catecholamine metabolite levels significantly decreased in a dose-dependent manner after metyrosine administration. Both systolic and diastolic blood pressure significantly decreased after metyrosine and surgical treatment. Metyrosine administration significantly improved insulin sensitivity, although surgery improved the the basal insulin secretion. Additionally, serum prolactin and thyroid-stimulatory hormone levels were significantly increased by metyrosine treatment, whereas plasma renin activity was decreased.

**Conclusions:**

Metyrosine significantly reduced catecholamines in patients with pheochromocytoma/paraganglioma and ensured the safety of the surgery. Adjustment of metyrosine administration may make surgical pretreatment more effective in achieving stabilized blood pressure and improving glucose metabolism. Endocrine parameters may manifest as the systemic effects of metyrosine administration.

## Introduction

Pheochromocytoma/paraganglioma (PPGL) is a neuroendocrine tumor that overproduces and secretes catecholamines including adrenaline, noradrenaline, and dopamine. Approximately 80–85% of these lesions arise from the adrenal medulla (pheochromocytoma), whereas 15–20% arise from the sympathetic ganglia (paraganglioma) (Lenders *et al.* 2014, Farrugia & Charalampopoulos 2019). PPGLs are associated with hypertension (85%) and glucose impairment (25–75%) (Wiesner *et al.* 2003, Pappachan *et al.* 2018, Farrugia & Charalampopoulos 2019). Interventions are required in patients with PPGL to prevent cardiovascular events attributable to high serum catecholamine levels (Fang *et al.* 2020, Y-Hassan & Falhammar 2020), although some previous studies did not recommend preoperative treatment (Groeben *et al.* 2017, Neumann *et al.* 2019). Furthermore, surgical removal of PPGL is recommended as the first-line treatment; however, certain PPGL lesions are unresectable owing to their multifocal or malignant nature (Lenders *et al.* 2014, Farrugia & Charalampopoulos 2019). In these situations, medication is required to compensate for reduced circulatory volume and improve cardiac dysfunction (Lenders *et al.* 2014, Farrugia & Charalampopoulos 2019).

Suppression of α1-adrenergic receptor signaling is required to ameliorate hyperactivity associated with high plasma catecholamine levels and refill the circulating plasma volume (Pacak 2007, Lenders *et al.* 2014, Farrugia & Charalampopoulos 2019, Fang *et al.* 2020). Metyrosine (alpha-methyl-para-tyrosine (αMPT)) has been used globally along with α-adrenergic blockers in patients with PPGL (Naruse *et al.* 2018, Gruber *et al.* 2021). In cases in which α1-adrenergic blockers are ineffective, insufficient, or difficult to use, αMPT is beneficial for reducing catecholamine levels and preventing comorbidities (Pacak 2007, Naruse *et al.* 2018, Fang *et al.* 2020). Furthermore, αMPT was effective in reducing total metanephrine (MN) levels by 50% in 31.3% of patients at a dose of approximately 1000 mg/day (Naruse *et al.* 2018). Additionally, αMPT has been suggested to be effective in improving hypertension and glucose intolerance associated with PPGL (Naruse *et al.* 2018). Regarding glucose metabolism, 43% of patients with PPGL developed hypoglycemia (plasma glucose level < 70 mg/dL) after surgical resection (Araki *et al.* 2019). High preoperative urinary MN levels have been identified as a risk factor for postoperative hypoglycemia (Chen *et al.* 2014, Araki *et al.* 2019). Thus, the suppression of preoperative catecholamine production by αMPT is considered effective for preventing rapid changes in α- and β-adrenergic receptor activity and subsequent postoperative hypoglycemia (Chen *et al.* 2014).

However, previous clinical studies (Engelman *et al.* 1968*a*,*b*, Nasrallah *et al.* 1977, Nicoletti *et al.* 1986, Perry *et al.* 1990, Sand *et al.* 1997, Steinsapir *et al.* 1997, Zimmermann *et al.* 2001, Wachtel *et al.* 2015, Butz *et al.* 2017, Hamidi *et al.* 2017, Naruse *et al.* 2018, Takekoshi *et al.* 2019) did not assess the effects of αMPT on glucose metabolism (Supplementary Table 1, see section on supplementary materials given at the end of this article). Additionally, whether αMPT administration enables safe surgical treatment remains unclear, even though αMPT is indicated to effectively reduce MN and normetanephrine (NMN) levels. The dose dependency of the effects of αMPT on catecholamine production and PPGL complications versus surgery also remains unclear.

Therefore, we conducted a retrospective cohort study at our university hospital to confirm the effectiveness of the αMPT administration on patients with PPGL. In this study, we focused on the effects of the drug on catecholamine levels, metabolic parameters including blood and physiological variables, and endocrinological functions. We compared these parameters before and after αMPT administration and after surgery to determine the effects of treatment with αMPT and surgery. We believe that this study will improve the prognosis of patients with PPGL and support further investigations of the pathophysiological problems associated with PPGL.

## Materials and methods

### Study design and inclusion criteria

**Figure 1:**
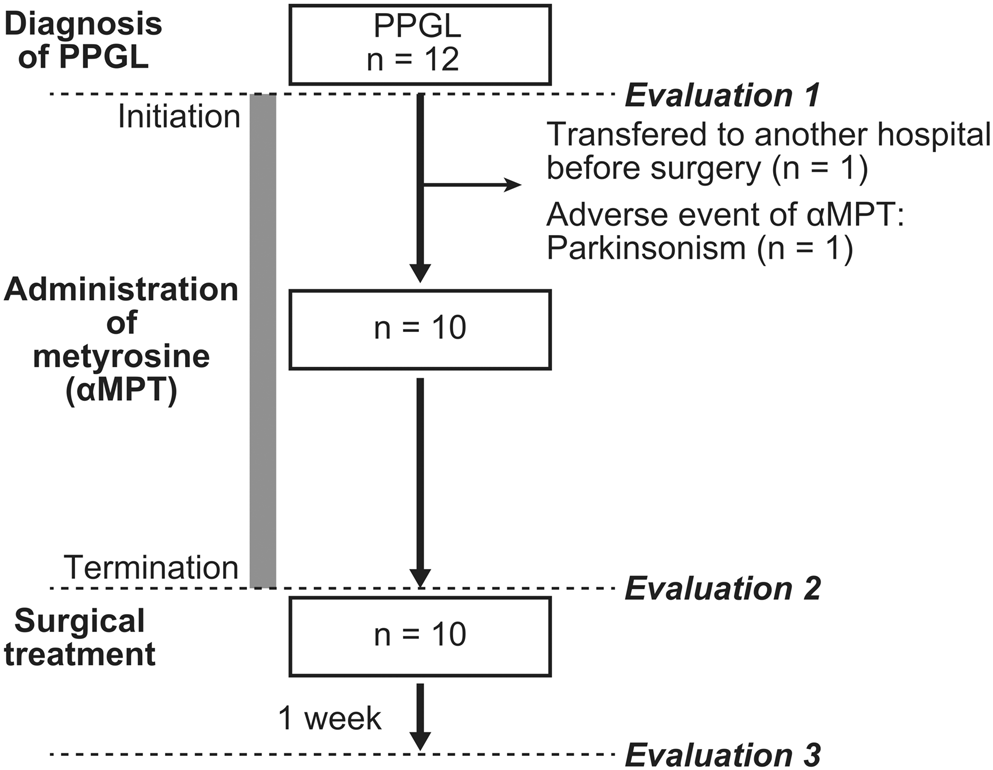
Flowchart of this study. The duration of metyrosine administration (26.7 ± 14.2 days) is indicated by a gray bar.

This retrospective, single-center cohort study evaluated patients with PPGL who were treated at Kurume University Hospital (Kurume City, Fukuoka, Japan) and registered in a clinical trial with the University Hospital Medical Information Network (UMIN) Center: UMIN000047430. As presented in the study flowchart (Fig. 1), we enrolled all 12 patients aged 20–90 years who were admitted to our hospital consecutively between May 1, 2018, and December 31, 2020. Finally, we included ten patients who underwent surgery after excluding two patients who did not at our hospital. The clinical diagnosis of PPGL was confirmed if they fulfilled the following criteria (Lenders *et al.* 2014, van Berkel *et al.* 2014, Neumann *et al.* 2019):

(i) Clinical symptoms of catecholamine excess (e.g. hypertension, glucose metabolism disorders, palpitations, sweating, and headache); (ii) elevations in 24-h urinary metanephrine fraction levels (>3 times the upper limit of the normal range); and (iii) detection of tumors suggestive of PPGL (CT imaging or MRI).

Additionally, we observed accumulation of ^123^I-metaiodobenzylguanidine in the tumors of all the patients in this study. Furthermore, all the patients who underwent surgery were histologically confirmed to have PPGL, although genetic testing was not performed. Evaluations were performed on ten patients before the initiation of metyrosine (Evaluation 1 in Fig. 1). Clinical findings, including age, sex, type of PPGL, body mass index (BMI), hemoglobin A1c (HbA1c) levels, and outcomes, are presented in Table 1. All patients were administered αMPT together with an α1-blocker that had been previously initiated and were included in this study. The clinical findings of the patients were evaluated before and after αMPT administration (Evaluation 2 in Fig. 1) and 1 week after surgical treatment (Evaluation 3 in Fig. 1).

**Table 1:** Patient background data, maximum metyrosine (αMPT) dose.

Case no.	Age, years	Sex	Type of PPGL, location^a^	BMI, kg/m^2^	HbA1c, % (NGSP)	Urinary metabolites of catecholamine			Administration of αMPT		Administration of doxazosin	
						MN + NMN, mg/day	MN, mg/day	NMN, mg/day	Maximum dose, mg/day	Duration, days	Maximum dose, mg/day	Duration, days
MPT-1	66	F	PC, left	29.2	7.6	1.12	0.65	0.47	750	27	2.0	27
MPT-2	57	F	PC, left	26.0	6.9	1.41	0.95	0.46	500	8	8.0	54
MPT-3	43	M	PC, bilateral	20.9	5.8	3.85	2.20	1.65	500	20	8.0	22
MPT-4	52	F	PC, right	19.1	6.6	3.98	2.72	1.26	500	8	3.0	13
MPT-5	66	M	PC, right	24.1	6.1	1.44	0.16	1.28	750	38	6.0	39
MPT-6	31	F	PC, left	16.2	6.5	6.18	0.10	6.08	1000	43	6.0	49
MPT-7	85	M	PGL, periabdominal aorta	24.1	6.2	1.03	0.08	0.95	750	13	3.0	63
MPT-8	42	M	PC, right	23.6	5.9	3.70	0.18	3.52	750	32	6.0	65
MPT-9	37	F	PC, left	16.7	6.2	33.40	12.50	20.90	1750	48	6.0	43
MPT-10	52	F	PC, left	19.9	5.3	4.90	2.94	1.96	1000	30	6.0	60
**Overall**	53.1 ±16.1^b^	M (4), F (6)	Right (3), left (5), bilateral (1), PGL (1)	22.0 ± 4.1^b^	6.3 ± 0.6^b^	3.78 (1.34–5.22)^c^	0.80 (0.15–2.78)^c^	1.47 (0.83–4.16)^c^	750 (500–1000)^c^	26.7 ±14.2^b^	5.4 ± 2.1^b^	43.5 ± 18.1^b^

^a^Laterality for PC and location for PGL are presented. ^b^Values are presented as the mean ± s.d.^c^Values are presented as median (interquartile range).

αMPT, metyrosine (α-methyl-para-tyrosine); BMI, body mass index; HbA1c, hemoglobin A1c; GSP, National Glycohemoglobin Standardization Program; MN, metanephrine; NMN, normetanephrine; PC, pheochromocytoma; PGL, paraganglioma.

### Administration of αMPT

The initial dose of αMPT was 500 mg/day in nine patients, whereas the remaining patient (MPT-7) started with a dose of 250 mg/day because of his advanced age (85 years) (Table 1). The αMPT dosage was maintained for more than three days in every patient, and urinary MN and NMN levels were evaluated. The dose was increased by 250–500 mg/day to achieve the goal of reducing urinary MN + NMN levels by more than 50% compared to pretreatment levels, dividing the dose between once and four times per day. Adherence to αMPT treatment was deemed sufficient (100% overall) during the study in all patients.

### Measurement of catecholamine metabolites

Urinary MN and NMN levels were measured by liquid chromatography/mass spectrometry (SRL, Tokyo, Japan) using the acid Uri Measure-T by collecting total urine for 24 h in accordance with the manufacturer’s protocol (Kanto Kagaku, Tokyo, Japan).

### Physiological and metabolic parameters

Physiological findings, including body height, body weight, BMI, and systolic and diastolic blood pressures, were regularly evaluated. Systolic and diastolic blood pressure and pulse rate were measured thrice in the sitting position. The average values of them were used for this study. Homeostasis model assessment of insulin resistance (HOMA-R) and beta-cell function (HOMA-β) as well as ΔC-peptide index (ΔCPI) were evaluated as metabolic parameters (Wallace *et al.* 2004, Saisho *et al.* 2011). HOMA-R was calculated as fasting insulin concentration (μU/mL) × fasting blood glucose level (mg/dL)/405 (Wallace *et al.* 2004). HOMA-β was calculated as fasting insulin concentration (μU/mL) × 360/(fasting blood glucose level (mg/dL) – 63) (Wallace *et al.* 2004). For ΔCPI, CPI (serum C-peptide (ng/mL)/plasma glucose level (mg/dL) × 100) (Saisho *et al.* 2011) was calculated before breakfast and 2 h after breakfast, and the difference was indicated as ΔCPI.

### Evaluation of endocrine function

Regarding endocrine function, prolactin, thyroid-stimulating hormone (TSH), free thyroxine (FT4), growth hormone (GH), insulin-like growth factor 1 (IGF1) levels, plasma renin activity (PRA), plasma aldosterone concentration (PAC), and aldosterone-to-renin ratio (ARR) were measured after overnight fasting in the early morning after lying in bed for 30 min.

### Evaluation of blood pressure and intervention for adjusting blood pressure during the perioperative period

Variations in systolic and diastolic blood pressure were evaluated according to the operative records of ten patients who underwent surgery. Additionally, transfusion volume, urinary volume, bleeding volume, phentolamine dose (mg), and duration (days) of catecholamine use were investigated.

### Ethical issues

All procedures were performed in accordance with the ethical standards of the Institutional Review Board of the Kurume University School of Medicine and the principles of the Declaration of Helsinki 2013. The requirement for written informed consent was waived for this retrospective study by the institutional Review Board of Kurume University School of Medicine. Instead, it was mandatory to disclose the research content on the Kurume University Hospital website. This study was approved by the Ethics Committee of the Kurume University School of Medicine (approval number: 20156).

### Statistical analysis

Quantitative data are presented as mean ± s.d. for normally distributed variables and as median plus interquartile range (IQR) for non-normally distributed variables. When normally distributed variables were compared with non-normally distributed variables, they were presented as medians plus IQR. The normal distribution of each variable was confirmed using the Shapiro–Wilk test. Qualitative data are expressed as proportions or percentages. To compare the means in groups of paired data, the Student’s *t*-test was applied for normally distributed variables, and Wilcoxon’s signed-rank test was used for non-normally distributed variables. All statistical analyses were performed using JMP^®^ 16 software (SAS Institute Inc., Cary, NC, USA). Statistical significance was set at *P* < 0.05.

## Results

### Baseline characteristics of the participants

The baseline characteristics of the ten participants are presented in Table 1. The mean age of the patients was 53.1 ± 16.1 years (95% confidence interval (CI) = 41.6–64.6). The participants included four men and six women. Of the ten patients, nine had pheochromocytoma, and one had paraganglioma (MPT-7). Of the pheochromocytomas, eight were unilateral (three on the right and five on the left) and one was bilateral (MPT-3). The mean BMI of the patients was 22.0 ± 4.1 kg/m^2^ (95% CI = 19.0–24.9). The mean HbA1c level (National Glycohemoglobin Standardization Program) of the patients was 6.3% ± 0.6% (95% CI = 5.9–6.8), and one patient (MPT-1) was taking oral hypoglycemic agents. The pretreatment with α1-blockade was performed in all patients preoperatively using doxazosin for 43.5 ± 18.1 days (maximum dose, 5.4 ± 2.1 mg/day) (Table 1).

Surgical treatment was performed consecutively, and αMPT administration was discontinued on the day of surgery (Fig. 1 and Table 1).

### Changes of catecholamine levels

**Figure 2:**
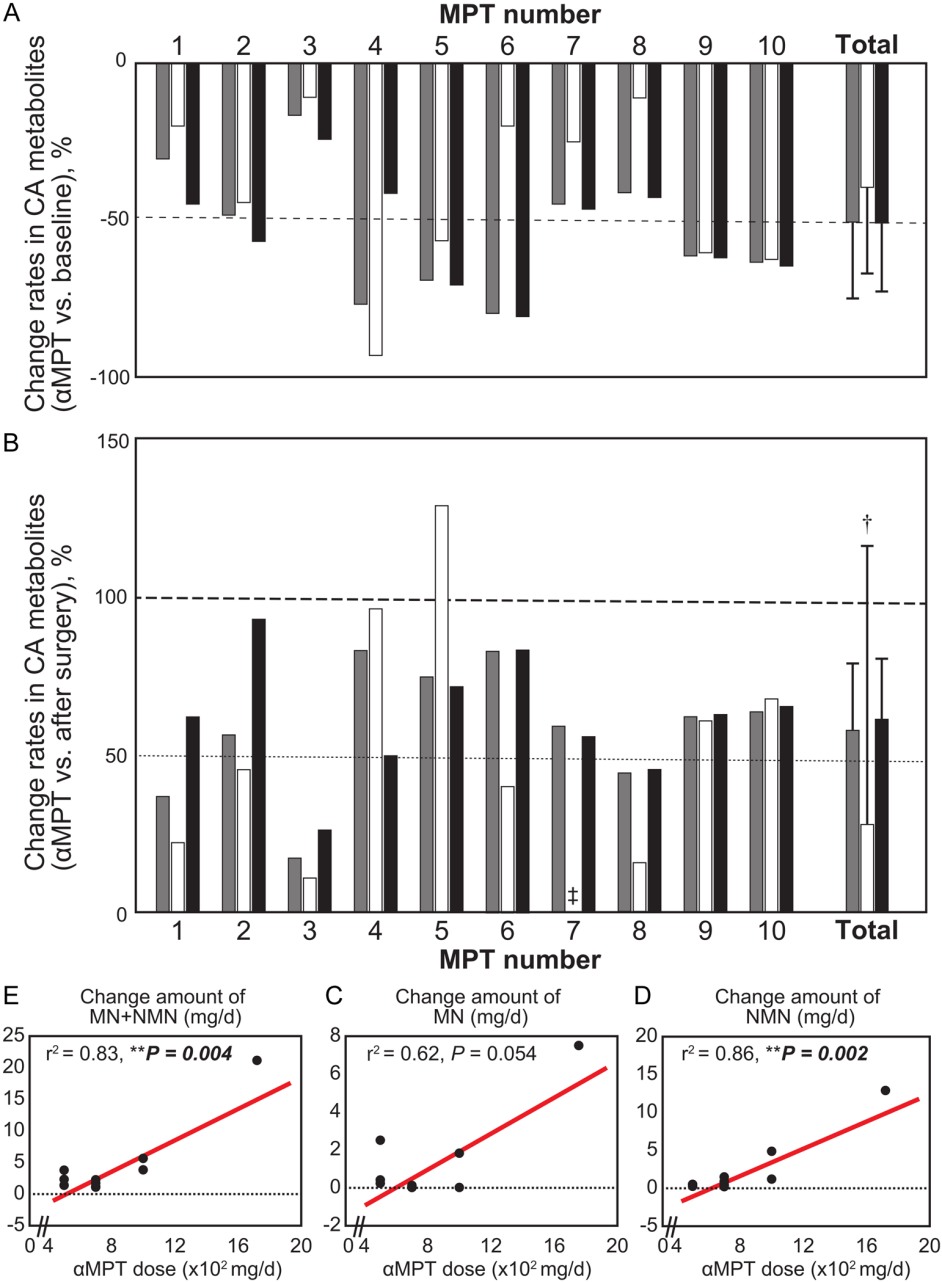
Changes in MN+NMN, MN, and NMN levels after αMPT administration and after surgery. (A) Differences between urinary catecholamine (CA) metabolite levels at baseline and after αMPT administration. (B) Differences between CA metabolite levels after αMPT administration and after surgical treatment. Changes in catecholamine metabolite levels between baseline and after αMPT administration are presented, comparing those between baseline and after surgical treatment as follows: CA metabolite levels (baseline − after αMPT administration)/CA metabolite levels (baseline – after surgery)) × 100%. †MN levels were not normally distributed and the median (IQR) was 42.6 (14.3–71.5). ‡Differences between baseline urinary MN levels at and MN levels after αMPT administration and after surgery are not presented because there was no postoperative decrease in MN levels. The urinary MN levels at baseline, after MPT administration, and after surgery were all low, with median levels of 0.08 mg/day, 0.06 mg/day, and 0.09 mg/day, respectively. For each participant, the MN + NMN, MN, and NMN levels are shown as gray, open, and closed bars, respectively. The error bars indicate the standard deviations. Correlations between the dose of αMPT and each catecholamine level are presented; (C) MN + NMN, (D) MN, and (E) NMN. Abbreviations: αMPT, α-methyl-para-tyrosine (metyrosine); CA, catecholamine; IQR, interquartile range; MN, metanephrine; NA, not applicable; NMN, normetanephrine.

The baseline levels of urinary MN + NMN, MN, and NMN, and the maximum dosage and periods of αMPT administration for each patient are presented in Table 1. The median maximum dose of αMPT for each patient was 750 mg/day (IQR = 500–1000 mg/day; range = 500–1750 mg/day). The mean duration of αMPT administration was 26.7 ± 14.2 days. Changes in urinary MN + NMN, MN, and NMN levels before and after αMPT administration and after surgical treatment are presented in Figs. 2A and B, respectively. The median urinary MN + NMN levels significantly decreased after αMPT administration (mean change rate = −53.0% ± 20.3%, 95% CI = −67.5 to −38.4, *P* = 0.001). Median urinary MN levels significantly decreased after αMPT administration (mean change rate = −40.2% ± 27.2%; 95% CI = −59.7 to −20.8, *P* = 0.001). The median urinary NMN levels significantly decreased after αMPT administration (mean change rate = −53.2% ± 16.5%; 95% CI = −65.8 to −41.4, *P* = 0.001). The dose of αMPT significantly correlated with the change levels of MN + NMN (*r*^2^ = 0.83, *P* = 0.004), MN (*r*^2^ = 0.62, *P* = 0.054), and NMN (*r*^2^ = 0.86, *P* = 0.002). Thus, catecholamine levels were significantly reduced by αMPT administration in a dose-dependent manner (Fig. 2C, D, and E).

### Physiological and metabolic parameters

**Figure 3:**
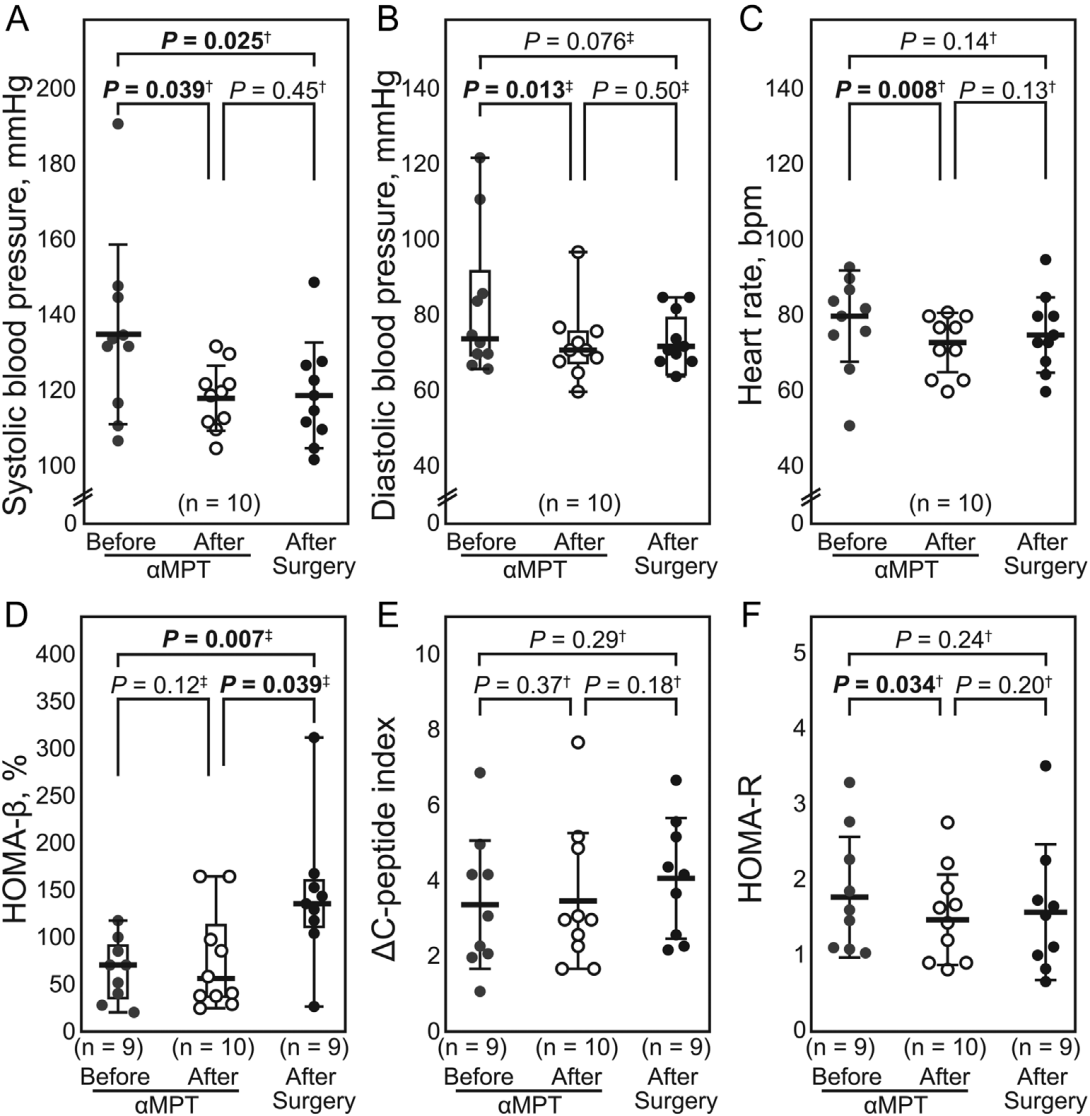
Changes in physiological and metabolic parameters after metyrosine (αMPT) administration and after surgical treatment. Normally distributed variables are presented as mean ± s.d. Non-normally distributed variables are presented as median (IQR (open box) and range (bars)). When normally and non-normally distributed variables were compared, they were presented as medians (IQR (open box) and range (bar)). †Data were analyzed using paired *t-test*. ‡Data were analyzed using Wilcoxon’s signed-rank test. *P* < 0.05 were considered significant and are presented in bold font. αMPT, α-methyl-para-tyrosine (metyrosine); bpm, beats per min; HOMA-β, homeostasis model assessment of β-cell function; HOMA-R, homeostasis model assessment of insulin resistance; NA, not applicable.

Changes in blood pressure and heart rate after αMPT administration are presented in Figs. 3A, B, and C, Table 2, and Supplementary Table 2. The mean systolic blood pressure changed by −12.5 mmHg (IQR = −18.3 to −2.3 (*n* = 10), *P* = 0.039). The median diastolic blood pressure changed by −7.5 mmHg (IQR = −11.0 to 0.3 (*n* = 10), *P* = 0.014). The heart rate also decreased significantly after αMPT administration (mean change = −6.1 beats per min (*n* = 10), 95% CI = −10.8 to −1.4, *P* = 0.008). No significant differences of the change levels in systolic blood pressure, diastolic blood pressure, or heart rate between the baseline measures and either after αMPT administration or postoperatively was observed (Table 2 and Figs. 3A, B, and C).

**Table 2:** Change levels before and after treatment.

Variables	Change levels (vs before αMPT administration)		*P*^b^
	After αMPT administration	After surgery	
**Blood pressure, mmHg (*n* = 10)**			
Systolic	−12.5 (IQR = −18.3 to −2.3)	16.0 (IQR = −29.0 to 1.75)^a^	0.89^c^
Diastolic	−7.5 (IQR = −11.0 to 0.3)	−8.0 (IQR = −20.0 to 4.5)^a^	>0.99^c^
**Heart rate, bpm (*n* = 10)**	−6.1 ± 6.5	−3.2 ± 8.9	0.26^d^
**Parameters for glucose metabolism (*n* = 8)**			
HOMA-β	11.4 ± 30.1	57.9 ± 42.5	**0.042** ^d^
ΔCPI	0.65 (IQR = −0.48 to 0.95)	0.40 (IQR = −0.95 to 1.07)^a^	0.74^c^
HOMA-R	−0.40 ± 0.64	−0.18 ± 0.70	0.41^d^

Normally distributed variables and non-normally distributed variables are presented as the mean ± s.d. and the median (interquartile range), respectively. ^a^Normally distributed variables; however, presented as median (interquartile range) to compare with non-normal distributed variables.^ b^*P* for significance between two groups (before to after metyrosine (αMPT) administration) and (before treatment to after surgery). ^c^*P*-values were analyzed using the Wilcoxon’s signed-rank test. ^d^*P*-values were analyzed using a paired *t*-test. *P*-values of <0.05 are considered significant (bold).

αMPT, α-methyl-para-tyrosine (metyrosine); bpm, beats per min; CPI, C-peptide index; HOMA-β, homeostasis model assessment of β-cell function; HOMA-R, homeostasis model assessment of insulin resistance.

The HOMA-β, ΔCPI, and HOMA-R values are presented in Figs. 3D, E, and F. Of the ten patients, eight (except for two patients (MPT-3 and 9) because of deficit data) were examined consecutively before αMPT administration and after surgery. Changes in the insulin secretory capacity after αMPT administration and surgical treatment are presented in Table 2, Figs. 3D and E, and Supplementary Table 3. αMPT administration did not change HOMA-β significantly; however, surgical treatment increased it significantly, when the mean change levels in HOMA-β were compared (αMPT vs surgery: 11.4% ± 30.1% vs 57.9% ± 42.5% (*n* = 8), *P* = 0.042). Regarding CPI, neither αMPT administration nor surgery significantly changed the ΔCPI values.

Changes in the patients’ insulin resistance after αMPT administration are shown in Table 2, Fig. 3F, and Supplementary Table 3. Although αMPT administration significantly decreased HOMA-R levels, surgery did not; however, no difference was observed between the medians of the two groups. Furthermore, no significant correlations were found between changes in HOMA-R and HOMA-β, and change levels in MN, NMN, or MN + NMN (Supplementary Fig. 1). However, ΔCPI decreased significantly after surgery but not after αMPT administration (Supplementary Fig. 2).

### Evaluation of endocrinological dysfunctions related to αMPT administration

Changes in endocrinological parameters after αMPT administration and surgical treatment are presented in Table 3 and Supplementary Table 4. The mean prolactin level was significantly increased by αMPT administration (*P* < 0.001) and surgery (*P* = 0.016). The median GH and IGF1 levels were not significantly altered by αMPT administration, although IGF1 levels significantly decreased after surgery (*P* = 0.007). Additionally, administration of αMPT significantly decreased PRA (*P* = 0.010), but not PAC (Table 3). Significant postoperative decreases were observed in both PRA (*P* = 0.022) and PAC (*P* = 0.038) (Table 3). Significant positive correlations were observed between the change levels in catecholamine metabolites (NMN and NMN + MN) and those in PRA after αMPT administration (Supplementary Fig. 3). Furthermore, both αMPT administration (*P* = 0.009) and surgery (*P* = 0.037) significantly increased ARR (Table 3). The median TSH level was significantly increased by αMPT administration (*P* = 0.024), whereas the mean FT4 level was not significantly altered (Table 3). Among the ten patients, one (MPT-7) was positive for anti-thyroid peroxidase antibody (25 IU/mL) despite normal thyroid function, and another patient (MPT-1) with Graves’ disease who was treated with thiamazole was positive for TSH receptor antibody (third generation, 3.7 IU/L; reference range < 2.0 IU/L).

**Table 3:** Changes of endocrinological parameters after metyrosine (αMPT) administration or surgical treatment.

Parameters	Pretreatment	After-αMPT	After surgery	αMPT/pretreatment^b^		Surgery/pretreatment^c^	
				Change rate, %	*P*	Change rate, %	*P*
Prolactin, ng/dL	12.7 (7.5–17.7)^d^ (*n* = 9)	39.4 (25.3–55.5)^d^ (*n* = 9)	17.4 (13.0–25.7) (*n* = 8)	251.7 ± 141.6	**<0.001**	46.4 (37.8–145.9)	**0.016**
TSH, μU/mL	0.90 (0.59–2.69) (*n* = 10)	1.54 (1.32–3.17) (*n* = 10)	2.16 (0.71–4.08)^a^ (*n* = 8)	72.0 ± 76.4	**0.024**	26.2 ± 64.8	0.37
Free thyroxine, ng/dL	1.06 ± 0.19 (*n* = 10)	1.05 ± 0.20 (*n* = 10)	1.17 ± 0.30 (*n* = 8)	−0.6 ± 9.9	0.43	2.6 (−2.3 to 30)	0.11
Growth hormone, ng/dL	0.47 (0.08–3.12) (*n* = 9)	0.37 (0.07–0.84)^a^ (*n* = 9)	0.48 (0.19–2.35) (*n* = 9)	−55.0 (−84.8 to 50.4)	0.098	149.5 ± 227.6	0.68
IGF1, s.d. value^d^	−1.2 ± 0.6 (*n* = 8)	−1.1 ± 0.7 (*n* = 8)	−1.6 ± 0.8 (*n* = 8)	0.1 ± 0.8	0.70	0.7 (−0.2 to 1.7)	**0.007**
PRA, ng/mL/h	1.1 (0.6–2.4)^a^ (*n* = 10)	0.4 (0.2–0.7) (*n* = 10)	0.3 (0.2–0.7) (*n* = 9)	−73.2 (−78.7 to −32.1)	**0.010**	−44.4 (−90.2 to −23.2)	**0.022**
PAC, pg/mL	140.4 ± 55.6 (*n* = 10)	102.3 ± 43.7 (*n* = 10)	90.4 ± 33.0 (*n* = 9)	−14.5 ± 52.8	0.93	−23.1 ± 34.5	**0.038**
ARR (PAC/PRA)	114 (88–184) (*n* = 10)	180 (120–574) (*n* = 10)	217 (100–681)^a^ (*n* = 9)	83.8 (31.5–192.5) (*n* = 10)	**0.009**	58.8 (−6.4 to 532.6) (*n* = 9)	**0.037**

Normally distributed and non-normally distributed variables are presented as the mean ± s.d. and the median (interquartile range), respectively. ^a^Normally distributed variables; however, presented as median (interquartile range) to compare with non-normal distributed variables. ^b^Change rates were calculated as (post-αMPT value – pre-αMPT value)/pre-αMPT value × 100%. ^c^Change rates were calculated as (postsurgery value – pre-αMPT value)/pre-αMPT value × 100%. ^d^IGF1 value is presented as s.d. value standardized by sex and age. Each value was analyzed using a paired* t*-test and Wilcoxon’s signed-rank test for normally and non-normally distributed variables, respectively. *P*-values of <0.05 are considered significant (bold).

αMPT, α-methyl-para-tyrosine (metyrosine); ARR, aldosterone-to-renin ratio; IGF1, insulin-like growth factor 1; PAC, plasma aldosterone concentration; PRA, plasma renin activity; PRL, prolactin; TSH, thyroid stimulation hormone

### Perioperative treatments of the patients

Infusion therapy was performed in each patient to manage their condition. Among the ten patients who underwent surgery, six patients (MPT-1, MPT-2, MPT-3, MPT-4, MPT-8, and MPT-9) required intraoperative phentolamine administration to manage blood pressure. Postoperative administration of inotropes was required in four patients, three of whom received intraoperative phentolamine. All patients who required >5 mg of phentolamine during surgical treatment used inotropes after tumor resection (Supplementary Table 5). No other adverse events, including cardiovascular issues, were observed in any patient during the perioperative period.

## Discussion

Administration of αMPT was effective in patients with PPGL, including those undergoing surgical treatment. Improvement of insulin sensitivity was observed after αMPT administration; however, suppressed insulin secretion could be recovered after surgery. Endocrinological changes attributable to decreased dopamine signals should be noted to prevent the resulting adverse events and estimate the efficacy of αMPT. Administration of the αMPT was indicated as an alternative treatment to pre-curative surgical treatment, although surgery is the best management.

Catecholamine levels were quickly reduced by the following αMPT therapy more than 50% compared with the reduction levels by surgery, and the changes in catecholamine levels positively correlated with the αMPT dose. This finding is in line with previous observations of 40–50% reductions following αMPT administration compared with the pretreatment level (Naruse *et al.* 2018). Additionally, both systolic and diastolic blood pressures were significantly reduced by αMPT administration, and blood pressure remained decreased during surgical treatment. Regarding variations in blood pressure, 30–60% of patients with PPGL have a risk of hypotension during and after surgery because preoperative exposure to catecholamines could result in the downregulation of their receptors (Gagner *et al.* 1996, Streeten & Anderson 1996, Kercher *et al.* 2002). In this context, preoperative αMPT administration was suggested to potentially reduce bleeding, anti-hypotensive/hypertensive agent use, and infusions during surgical treatment among patients with PPGL, versus the findings in patients who did not receive αMPT (Perry *et al.* 1990, Wachtel *et al.* 2015, Butz *et al.* 2017). Although preoperative suppression of catecholamine production by αMPT can also effectively prevent postoperative hypotension, we administered inotropes after surgery in 40% of the patients. Further investigation is required to determine the most effective protocol of combined presurgical αMPT, for a 50% reduction in catecholamine levels, and α-blocker treatment, for blood pressure management. The heart rate with αMPT was slower than that at baseline, although surgery did not demonstrate significant reduction compared with baseline. However, previous studies have shown significant reductions in the heart rate after surgery (Di Stolfo *et al.* 2019). In this context, evaluations of surgery on cardiovascular system, including heart rate and blood pressure, should be evaluated in larger study volume and after more than 1 week from surgery.

Surgery was more effective than αMPT administration in increasing insulin secretion, although αMPT administration increased insulin sensitivity. We could not find a significant difference in the HOMA-R values between baseline and post surgery. Surgical effects were considered a plausible reason, as the evaluation was performed 1 week postoperatively. Another plausible reason is the postoperative catecholamine administration (MP-4, MP-5, MP-8, and MP-9). Additionally, we could not exclude the false negatives based on the small number of the participants. Excessive catecholamine content was proven to reduce insulin secretion and sensitivity (Cryer 1993), although the mechanisms by which PPGL contributes to glucose impairment are complicated in each patient. Metabolome analysis of patients with PPGL before and after surgery indicated the effects of catecholamine excess on a wide range of metabolic disorders, including glucose impairment (Erlic *et al.* 2019, März *et al.* 2021). Activation of α2-adrenergic receptors reduces the insulin secretory capacity in islet cells (Abe *et al.* 2020). In contrast, activation of the β-adrenergic receptor suppresses glucose uptake via insulin signaling in skeletal muscles (Shiuchi *et al.* 2017). Additionally, β-adrenergic receptor signals enhance/promote glucagon secretion in pancreatic α-cells (Hamilton *et al.* 2018), lipolysis in adipose tissue (Abe *et al.* 2020, Fang *et al.*, 2020), and glycogenolysis and gluconeogenesis in the liver (Abe *et al.* 2020). In fact, euglycemic hyper-insulinemic clamping performed before and after surgery in patients with pheochromocytoma demonstrated that endogenous catecholamine excess induces insulin insensitivity even in patients with normal glucose tolerance (Wiesner *et al.* 2003). Furthermore, rebounds of insulin secretion and sensitivity levels after surgical removal of PPGL lesions should be noted to prevent critical outcomes. Decreased α- and β-adrenergic signals result in insulin hypersecretion and the recovery of insulin sensitivity, respectively, after surgical treatment, thereby increasing the risk of hypoglycemia (Chen *et al.* 2014). Although αMPT administration did not significantly improve insulin secretion, it was possibly effective in reducing postoperative gaps in insulin overactivity. Additionally, the patients in this study did not exhibit severe variations in their blood glucose profiles during surgical treatment. Therefore, αMPT administration in patients with PPGL may be effective for perioperative glycemic control, albeit with lower efficacy than surgical treatment and increase the safety of surgery by preventing postoperative hypoglycemia.

Examining endocrinological function is useful for preventing adverse events and estimating the efficacy of αMPT administration. Prolactin levels significantly increased after αMPT administration, possibly because of the suppression of dopamine production in the central nervous system, which is likely to be a dopamine receptor antagonist (Ajmal *et al.* 2014). In contrast to PRL, dopamine physiologically increases the GH and IGF1 levels (Díaz-Torga *et al.* 2020). However, we could not confirm any significant changes in the GH and IGF1 levels in this short-period study. High levels of PRA and PAC and low levels of ARR have been reported in patients with PPGL due to β1 stimulation and reduced circulatory plasma volume (Yamada *et al.* 2020). In contrast, the administration of αMPT significantly reduced PRA, which was followed by PAC reduction and ARR elevation. Moreover, the preoperative recommendation of salt intake to maintain fluid volume may have contributed to the decrease in the PRA and PAC. Thus, evaluations of PRA, PAC, and ARR may be good indicators of effective premedication using the αMPT. Dopamine suppresses TRH, which leads to thyroid dysfunction manifested as low TSH and fT4 levels (Mohammadi *et al.* 2021). Thus, TSH levels were significantly elevated after αMPT administration (Nicoletti *et al.* 1986), probably through inhibition of dopamine production in this study. Therefore, increases in PRL and TSH levels may contribute to preventing the progression of adverse events, that is, they may serve as surrogate markers of the systemic effects of αMPT.

This study had two limitations. First, this study did not include a control group without αMPT administration because PPGL is a lethal disease and maximum caution should be exerted. Second, a small number of patients were examined in this study, because PPGL is a rare disease. However, we believe that future large-scale randomized controlled prospective trials will confirm our conclusions.

## Conclusion

Administration of αMPT reduced catecholamine levels to 1/2 in patients with PPGL who were preparing for surgical treatment in this study period. Glucose impairment was partially improved by αMPT administration, although surgery improved insulin secretion and sensitivity depending on the reduction in catecholamine levels. Endocrinological changes attributable to decreased dopamine signals should be monitored to prevent the resulting adverse events and to assess the effectiveness of αMPT. In patients who cannot tolerate α-adrenergic blockade (including persistent or orthostatic hypotension), have persistent hypertension despite sufficient α-adrenergic blockade, or require surgery or other treatments that may release large amounts of catecholamines, the αMPT administration was suggested to stabilize blood pressure and glucose metabolism and ensure the safety of surgery and improve the prognosis.

## Supplementary Materials

Supplementary Tables

Supplementary Figure 1

Supplementary Figure 2

Supplementary Figure 3

## Declaration of interest

The authors declare that there is no conflict of interest that could be perceived as prejudicing the impartiality of the research reported.

## Funding

This study did not receive any specific grants from any funding agency in the public, commercial, or not-for-profit sector.

## Author contribution statement

YM and KA designed the study, and contributed equally. YM, KA, KM, and MG collected the data. YM, KA, AN, JY, SI, TI, and MN interpreted the data. YM and KA drafted the first manuscript. AN, KM, MG, JY, NO, HK, KC, SI, YI, NH, SM, TI, and MN reviewed the manuscript. YM, KA, AN, TI, and MN revised the manuscript. YM, KA, AN, KM, MG, JY, NO, HK, KC, SI, YI, NH, SM, TI, and MN provided inputs for the preparation of the manuscript. All authors have read and approved the final version of the manuscript.
