# Supplementary material for: Metyrosine-associated endocrinological changes in pheochromocytoma and paraganglioma
Source: Endocr Oncol. 2023 Aug 30;3(1):e230006. doi: 10.1530/EO-23-0006 (PMC10563611; doi:10.1530/EO-23-0006)
Supplement: Supplementary Tables [file supplementary_tables.pdf]

Supplementary Table 1. Previous clinical studies using metyrosine, α-Methyl-para-tyrosine (αMPT), administration to the patients with pheochromocytoma/paraganglioma (PPGL)

No.	Author	Year	Primary outcome/Contents
**ST-1**	Takekoshi K, et al.	2019	Evaluation of the achievement of 50% reduction in uMN or uNMN or catecholamines from baseline levels
**ST-2**	Naruse M, et al.	2018	The proportion of patients who achieved 50% or more reduction in uMN or uNMN from baseline levels
**ST-3**	Butz JJ, et al	2017	Perioperative clinical course and complications of patients with PPGL w/wo αMPT administration
**ST-4**	Hamidi O, et al	2017	Baseline description, survival outcomes, and predictors of shorter survival evaluations in patients with rapidly progressive and indolent disease (Malignant pheochromocytoma)
**ST-5**	Wachtel H, et al	2015	Intraoperative hemodynamics, measured by heart rate and systolic blood pressure
**ST-6**	Zimmermann RC, et al	2001	Reduction of circadian rhythm in TSH levels and changes in prolactin levels
**ST-7**	Steinsapir J, et al	1997	The percentage of patients not requiring pressors or phentolamine during the intraoperative period and perioperative peak systolic pressures and peak heart rates in each group
**ST-8**	Sand J, et al	1997	Blood pressure, complications, and survival rate of the patients with PPGL after surgery w/wo preoperative αMPT
**ST-9**	Perry RR, et al	1990	Differences in maximum, minimum, or mean blood pressure before or after tumor resection w/wo αMPT
**ST-10**	Nicoletti I, et al	1986	Relationships between Catecholamine, and TSH or prolactin levels

**ST-11**	Nasrallah HA, et al	1977	Clinical improvement using the National Institute of Mental Health Inpatient Behavioral Rating Scale or the Brief Psychiatric Rating Scale (Schizophrenia)
**ST-12**	Engelman K, et al	1968	Inhibition of catecholamine synthesis and improvement of hypertension
**ST-13**	Engelman K, et al	1968	Identification and quantification of urinary products of the drug after oral dosing (Pharmacological study)

ST-1) Takekoshi K, et al. Endocr J. 2019;66(12):1063–1072. ST-2) Naruse M, et al. Endocr J. 2018;65(3):359–371. ST-3) Butz JJ, et al. Int J Surg. 2017;46:1–6. ST-4) Hamidi O, et al. J Clin Endocrinol Metab. 201;102(9):3296–3305. ST-5) Wachtel, et al. Ann Surg Oncol. 2015;22 Suppl 3:S646–S654. ST-6) Zimmermann RC, et al. J Soc Gynecol Investig. 2001;8(3):174–178. ST-7) Steinsapir J, et al. Arch Intern Med. 1997;157(8):901–906. ST-8) Sand J, et al. Ann Chir Gynaecol. 1997;86(3):230–232. ST-9) Perry RR, et al. Ann Surg. 1990;212(5):621–628. ST-10) Nicoletti I, et al. Acta Endocrinol (Copenh). 1986;111(2):154–161. ST-11) Nasrallah HA, et al. Arch Gen Psychiatry. 1977;34(6):649–655. ST-12) Engelman K, et al. J Clin Invest. 1968;47(3):577–594. ST-13) Engelman K, et al. J Clin Invest. 1968;47(3):568–576.

Abbreviations: αMPT, α-Methyl-para-tyrosine; PPGL, pheochromocytoma/paraganglioma; uMN, urinary metanephrine; uNMN, urinary normetanephrine.

Supplementary Table 2. Changes in blood pressures and heart rate of each patient and overall values

Case No.	Systolic blood pressure, mmHg			Diastolic blood pressure, mmHg			Heart rate, bpm		
	**Before**	**αMPT**	**P/S**	**Before**	**αMPT**	**P/S**	**Before**	**αMPT**	**P/S**
**MPT-1**	134	122	105	67	65	71	75	63	63
**MPT-2**	132	120	123	73	73	68	51	60	60
**MPT-3**	132	119	115	86	77	68	84	77	73
**MPT-4**	107	113	127	66	71	72	76	71	68
**MPT-5**	111	122	112	70	71	74	80	71	75
**MPT-6**	148	132	119	111	97	85	93	81	95
**MPT-7**	145	130	149	70	60	82	66	63	80
**MPT-8**	117	112	102	75	68	64	82	80	73
**MPT-9**	191	105	128	122	69	85	90	77	80
**MPT-10**	135	110	110	84	76	70	87	80	85
**Overall†** (n = 10)	135 ± 24	119 ± 9	119 ± 14	74 (69–92)	71 (67–76)‡	72 (68–83)‡	78 ± 12	72 ± 8	75 ± 10

†Values are presented as the mean ± standard deviation for normally distributed variables and as the median (interquartile range) for non-normally distributed variables. ‡Normally distributed variables; however, presented as median (interquartile range) to compare with non-normal distributed variables.

Abbreviations: αMPT, α-methyl-para-tyrosine (metyrosine); bpm, beats per minute; NA, not applicable.

Supplementary Table 3. Changes in parameters related to glucose metabolism before and after metyrosine (αMPT) administration and surgical treatment of each patient

Case No.	HOMA-β			ΔC-peptide index			HOMA-R		
	Before	αMPT	P/S	Before	αMPT	P/S	Before	αMPT	P/S
**MPT-1**	53.2	59.7	137.0	2.0	3.0	2.6	3.32	1.92	1.76
**MPT-2**	101.5	166.0	145.0	2.1	1.7	2.2	2.80	1.66	3.54
**MPT-3**	72.0	99.0	NA	2.3	3.0	NA	2.30	1.70	NA
**MPT-4**	29.4	26.2	105.6	6.9	7.7	5.6	1.49	0.84	0.85
**MPT-5**	72.0	42.1	130.9	4.2	5.2	4.4	1.63	1.23	1.68
**MPT-6**	21.8	30.4	154.3	3.1	2.6	4.2	1.13	1.46	1.14
**MPT-7**	41.8	39.1	28.0	1.1	1.7	2.3	1.06	0.93	0.68
**MPT-8**	119.1	165.9	169.0	4.2	4.9	5.2	1.88	2.25	2.29
**MPT-9**	NA	39.3	313.2	NA	3.1	6.7	NA	2.79	1.56
**MPT-10**	86.7	87.1	119.2	5.0	2.3	3.7	1.11	0.93	1.03
**Overall†**	72.0 (35.6–94.1)‡ (n = 9)	50.9 (36.9–115.7) (n = 10)	137.0 (112.4–161.7)‡ (n = 9)	3.4 ± 1.8 (n = 9)	3.5 ± 1.9 (n = 10)	4.1 ± 1.6 (n = 9)	1.9 ± 0.8 (n = 9)	1.6 ± 0.6 (n = 10)	1.6 ± 0.9 (n = 9)

†Normally distributed variables and non-normally distributed variables are presented as mean ± standard deviation and median (interquartile range), respectively. ‡Normally distributed variables; however, presented as median (interquartile range) to compare with non-normal distributed variables.

Abbreviations: αMPT, α-methyl-para-tyrosine (metyrosine); bpm, beats per minute; NA, not applicable; CPI, C-peptide index; HOMA-R, homeostasis model assessment of insulin resistance; HOMA-β, homeostasis model assessment of beta-cell function.

Supplementary Table 4. Changes of the endocrinological parameters in each patient

Case No.	PRL, ng/dL			TSH, µU/mL			FT4, pg/mL			GH, ng/dL			IGF-1, SD value‡			PRA, ng/mL/h			PAC, pg/mL		
	Pre	αMPT	P/S	Pre	αMPT	P/S	Pre	αMPT	P/S	Pre	αMPT	P/S	Pre	αMPT	P/S	Pre	αMPT	P/S	Pre	αMPT	P/S
**MPT-1**	4.8	25.7	11.9	0.60	1.52	NA	1.29	1.16	NA	0.06	0.03	0.14	−1.3	−2.1	−1.5	1.3	0.3	0.2	154.3	170.6	132.9
**MPT-2**	12.4	32.1	17.2	1.97	2.76	2.14	1.05	1.13	1.07	0.09	0.66	0.22	−0.2	−0.3	−1.6	0.3	1.0	0.8	55.8	106.9	70.4
**MPT-3**	18.5	14.9	NA	0.58	1.68	0.81	1.18	1.11	1.09	0.11	0.10	0.15	−1.5	−0.8	−0.8	0.7	0.2	0.3	76.0	50.0	65.2
**MPT-4**	22.5	69.8	NA	0.36	0.93	NA	0.96	0.87	NA	6.61	0.87	NA	−1.9	−1.5	NA	2.0	0.5	NA	188.3	90.0	NA
**MPT-5**	6.2	24.9	3.0	0.75	1.56	0.40	1.22	1.14	1.26	0.57	0.12	3.05	−0.3	−0.8	−0.8	2.8	2.0	1.9	114.1	176.8	123.0
**MPT-6**	8.8	47.3	59.5	0.89	1.47	2.17	0.79	0.87	1.09	0.47	0.80	0.58	−1.4	−0.8	−2.7	2.4	0.6	0.1	248.2	94.0	75.0
**MPT-7**	12.7	52.1	17.5	5.58	5.23	5.40	1.05	1.10	1.80	2.74	1.10	1.64	NA†	NA†	NA†	0.3	0.2	0.2	132.7	118.2	139.4
**MPT-8**	13.8	39.4	26.1	1.12	1.45	2.20	1.18	1.25	1.25	0.04	0.04	0.26	−1.2	−0.5	−1.0	0.7	0.1	0.6	128.4	94.1	67.7
**MPT-9**	NA	NA	16.2	4.83	4.41	4.71	0.72	0.61	0.70	NA	NA	8.85	NA	NA	−1.3	2.5	0.4	0.1	177.2	50.0	50.0
**MPT-10**	16.8	58.9	24.6	0.92	0.80	0.67	1.11	1.25	1.10	3.50	0.37	0.48	−1.8	−1.9	−3.0	0.9	0.4	0.5	129.4	72.1	90.4

†One patient (MPT-7), aged 85 years, was excluded from this analysis. ‡The IGF-1 value is presented as a standard deviation (SD), standardized for sex and age.

Abbreviations: αMPT, α-methyl-para-tyrosine (metyrosine); FT4, free thyroxine 4; GH, growth hormone; IGF-1, insulin-like growth factor-1; PAC, plasma aldosterone concentration; PRA, plasma renin activity; PRL, prolactin; SD, standard deviation; TSH, thyroid-stimulating hormone.

Supplementary Table 5. Perioperative treatment of patients who received metyrosine

Case no.	Infusion volume (mL)	Urine volume (mL)	Bleeding volume (mL)	Intraoperative phentolamine mesylate (mg)	Postoperative use of inotropes (days)
**MPT-1**	1570	500	6	1.5	None
**MPT-2**	1650	400	5	0.5	None
**MPT-3**	2670	500	15	4	None
**MPT-4**	4005	1400	35	5	Required (2)†
**MPT-5**	1640	150	22	0	Required (5)†
**MPT-6**	1770	300	102	0	None
**MPT-7**	2270	500	55	0	None
**MPT-8**	1450	900	113	12	Required (4)†
**MPT-9**	2500	1000	103	30	Required (1)†
**MPT-10**	1200	650	5	0	None
**Overall**	2073 ± 828	630 ± 372	28.5 (5.8–102.3)	1.0 (0.0–6.8)	

†Inotropes were used after surgical treatment. Durations of inotropes administration (in days) are presented in parentheses.

Abbreviation: NA, not applicable.
