# Supplementary material for: Metyrosine-associated endocrinological changes in pheochromocytoma and paraganglioma
Source: Endocr Oncol. 2023 Aug 30;3(1):e230006. doi: 10.1530/EO-23-0006 (PMC10563611; doi:10.1530/EO-23-0006)

Supplementary Figure 1. Correlations between change levels in catecholamine metabolite, and insulin secretion or sensitivity; Between before and after metyrosine administration

Correlations between changes in HOMA-β and reductions in (A) Urinary MN+NMN, (B) Urinary MN, and (C) Urinary NMN levels after αMPT administration are presented. Correlations between changes in ΔC-peptide index and reductions in (D) Urinary MN+NMN, (E) Urinary MN, and (F) Urinary NMN levels, and correlations between changes in HOMA-R and reductions in (G) Urinary MN+NMN, (H) Urinary MN, and (I) Urinary NMN levels. None of these correlations were significant (n = 8). MPT-3 and MPT-9 did not undergo HOMA-β, ΔC-peptide index, and HOMA-R evaluations (deficit data).
The open circles represent each patient’ s value. MPT-1, blue; MPT-2, green; MPT-3, N.A.; MPT-4, sky blue; MPT-5, magenta; MPT-6, brown; MPT-7, orange; MPT-8, purple; MPT-9, N.A.; MPT-10, black.
Abbreviations: αMPT, α-methyl-para-tyrosine (metyrosine); HOMA-β, homeostasis model assessment of β-cell function; HOMA-R, homeostasis model assessment of insulin resistance; MN, metanephrine; NMN, normetanephrine.

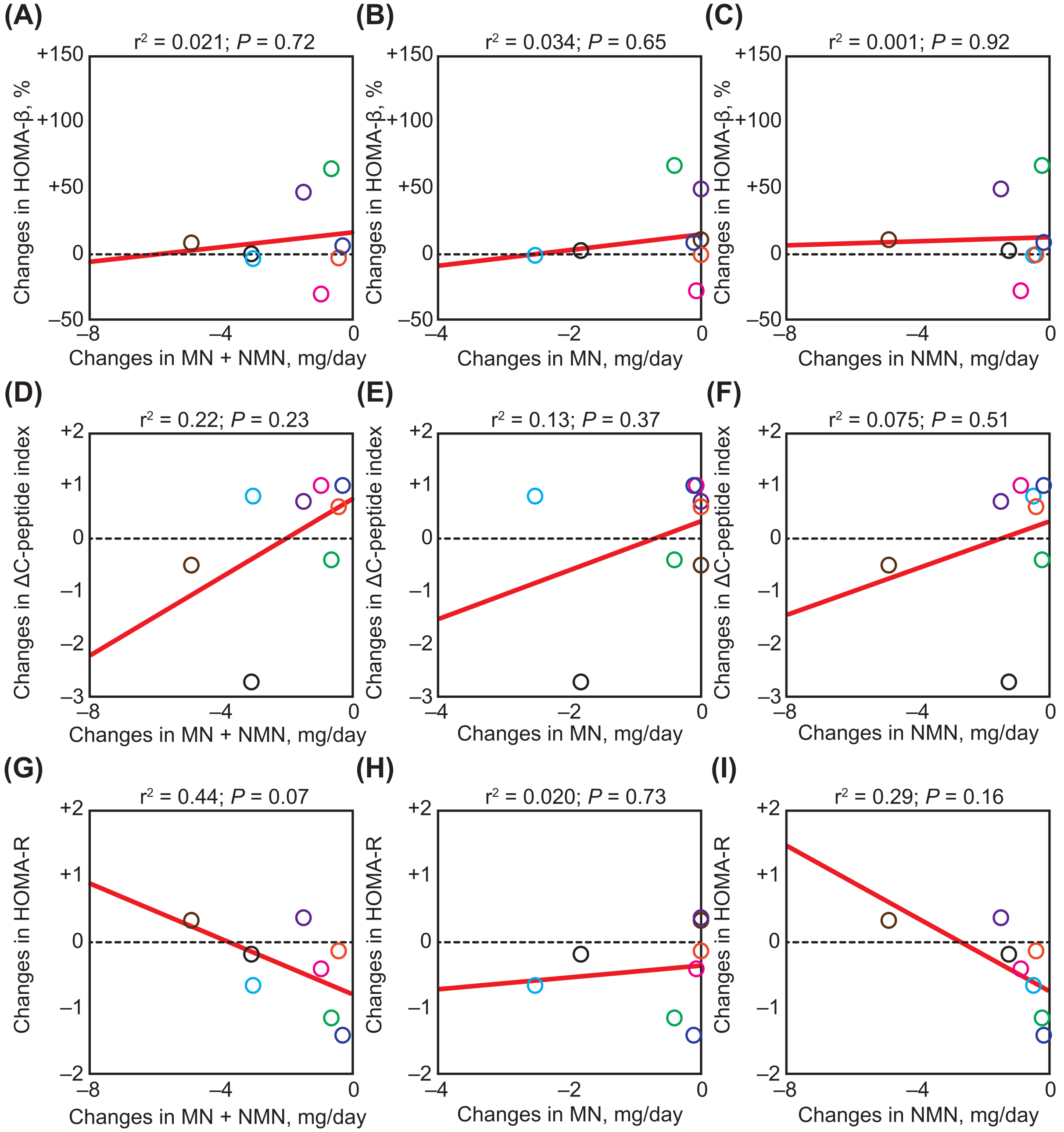

Supplement: Supplementary Figure 1 [file supplementary_figure_1.pdf]
