# Supplementary material for: Metyrosine-associated endocrinological changes in pheochromocytoma and paraganglioma
Source: Endocr Oncol. 2023 Aug 30;3(1):e230006. doi: 10.1530/EO-23-0006 (PMC10563611; doi:10.1530/EO-23-0006)

Supplementary Figure 3. Correlations between change levels in catecholamine metabolite, and plasma renin activity, plasma aldosterone concentration, and aldosterone-to-renin ratio; Between before and after metyrosine administration

Correlations between changes in plasma renin activity and reductions in (A) Urinary MN+NMN, (B) Urinary MN, and (C) Urinary NMN levels after αMPT administration are presented. Correlations between changes in plasma aldosterone concentration and reductions in (D) Urinary MN+NMN, (E) Urinary MN, and (F) Urinary NMN levels, and correlations between changes in aldosterone-to-renin ratio and reductions in (G) Urinary MN+NMN, (H) Urinary MN, and (I) Urinary NMN levels. Both change levels in plasma renin activity and plasma aldosterone concentration significantly correlations with those in catecholamine metabolites (n = 10). The open circles represent each patient' s value. MPT-1, blue; MPT-2, green; MPT-3, lime green; MPT-4, sky blue; MPT-5, magenta; MPT-6, brown; MPT-7, orange; MPT-8, purple; MPT-9, grey; MPT-10, black. Abbreviations: αMPT, α-methyl-para-tyrosine (metyrosine); ARR, aldosterone-to renin ratio; MN, metanephrine; NMN, normetanephrine; PAC, plasma aldosterone concentration; PRA, plasa renin activity.

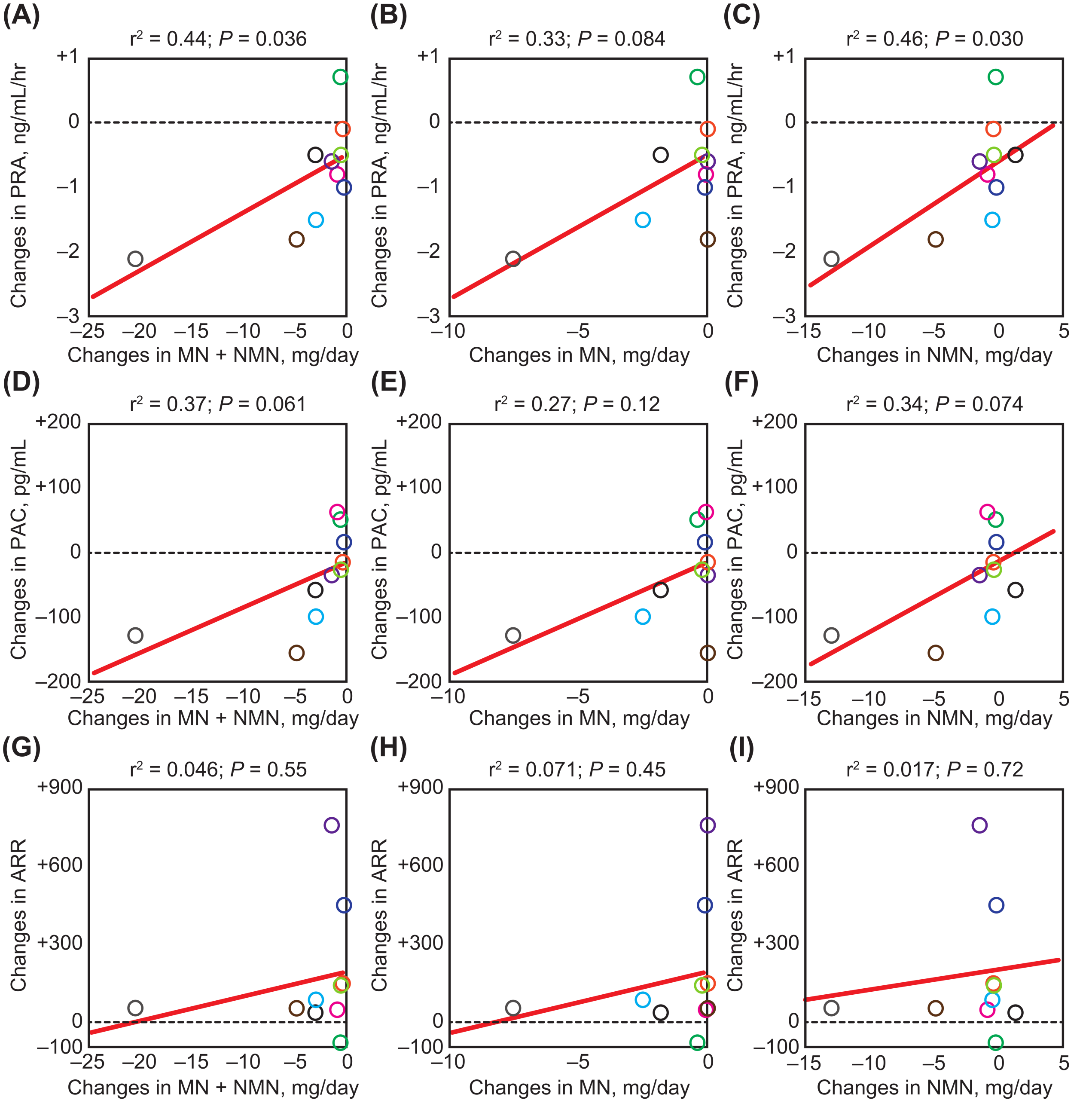

Supplement: Supplementary Figure 3 [file supplementary_figure_3.pdf]
